# Metabolic Proteins Expression Up-Regulated in Blood-Borne Extensively Drug-Resistant *Salmonella* Typhi Isolates from Pakistan

**DOI:** 10.3390/medicina60091404

**Published:** 2024-08-27

**Authors:** Nusrat Yasin, Hazir Rahman, Muhammad Qasim, Iqbal Nisa, Yasra Sarwar, Niamat Khan, Khalid J. Alzahrani, Meshari A. Alsuwat, Fuad M. Alzahrani, Abrar Aljohani

**Affiliations:** 1 Department of Microbiology, Kohat University of Science and Technology, Kohat 26000, Pakistan; nusratyasin47@gmail.com (N.Y.); qasim89@gmail.com (M.Q.); 2 Department of Microbiology, Abdul Wali Khan University Mardan, Mardan 23200, Pakistan; 3 Department of Microbiology, Women University Swabi, Swabi 23430, Pakistan; nisayam55@gmail.com; 4 Health Biotechnology Division, National Institute for Biotechnology and Genetic Engineering, Faisalabad 38000, Pakistan; yasrasarwar@yahoo.com; 5 Department of Biotechnology and Genetic Engineering, Kohat University of Science and Technology, Kohat 26000, Pakistan; niamat.khattak@gmail.com; 6 Department of Clinical Laboratories Sciences, College of Applied Medical Sciences, Taif University, P.O. Box 11099, Taif 21944, Saudi Arabia; ak.jamaan@tu.edu.sa (K.J.A.); m.alsawat@tu.edu.sa (M.A.A.); fuadmubarak@tu.edu.sa (F.M.A.);

**Keywords:** MDR and XDR *S*. Typhi, proteome, orbitrap MS analysis, functional and antigenic annotation

## Abstract

*Background and Objectives*: In the undertaken study, proteomics alterations of blood-borne XDR *S.* Typhi isolated from Pakistan were investigated using mass spectrometry. *Materials and Methods*: MDR and XDR *S.* Typhi total protein lysates were fractionated, digested, and processed for nanoflow LC-LTQ-*Orbitrap MS* analysis. *Results*: Among the 1267 identified proteins, 37 were differentially regulated, of which 28 were up-regulated, and 9 were down-regulated in XDR *S.* Typhi as compared to MDR *S.* Typhi. Based on the functional annotation, proteins found up-regulated are involved mainly in metabolic pathways (ManA, FadB, DacC, GpmA, AphA, PfkB, TalA, FbaB, OtsA, 16504242), the biosynthesis of secondary metabolites (ManA, FadB, GlpB, GpmA, PfkB, TalA, FbaB, OtsA), microbial metabolism in diverse environments (FadB, GpmA, PfkB, NfnB, TalA, FbaB), and ABC transporters (PstS, YbeJ, MglB, RbsB, ArtJ). Proteins found down-regulated are involved mainly in carbon metabolism (FadB, GpmA, PfkB, FalA, FbaB) and the biosynthesis of amino acids (GpmA, PfkB, TalA, FbaB). Most of the identified differential proteins were predicted to be antigenic, and matched with resistome data. *Conclusions*: A total of 28 proteins were up-regulated, and 9 were down-regulated in XDR *S.* Typhi. Further characterization of the identified proteins will help in understanding the molecular signaling involved in the emergence of XDR *S*. Typhi.

## 1. Introduction

*Salmonella enterica serovar* Typhi (*S.* Typhi) is a Gram-negative human pathogen that causes typhoid. The global data reflected almost 21 million people infected with *S.* Typhi. Among the South Asian countries, Pakistan has an estimated rate of 493.5 per 100,000 cases in 2018 [1].

The emergence of antimicrobial resistance (AMR), and even the augmented rate of extensively drug-resistant (XDR) *S.* Typhi, is a global threat to public health and leads to worse clinical outcomes, prolonged hospital stays, cost, and mortality rates [2,3]. Understanding the microbial resistance mechanism against the available antibiotics is the ultimate strategy for disease surveillance [4]. 

Proteomic techniques are currently in use for the elucidation of biomarker proteins, the identification of antigenic molecules, and the virulence markers of *S.* Typhi [5,6]. Proteomics is a rapidly emerging tool for identifying drug resistance pathways in infectious diseases caused by *S.* Typhi [7,8]. 

Previously, the molecular strain typing and drug resistance of *S.* Typhi from Pakistan were reported [9]; however, from literature mining, there is a lack of data on the differential proteome profile of XDR *S.* Typhi. This study aimed to investigate the proteome of blood-borne XDR *S.* Typhi hospital isolates in Pakistan. Findings from the work will be helpful in understanding the molecular mechanism leading to XDR *S.* Typhi.

## 2. Materials and Methods

### 2.1. Sampling

Blood samples from the suspected typhoid patients in tertiary care hospitals in Kohat, Islamabad, and Rawalpindi were collected and cultured. Informed patient consent was obtained, and ethical approval was granted by the KUST research ethics committee (Ref. No. KUST/Ethical Committee/16-14).

### 2.2. Isolation and Identification of S. Typhi

Clinical samples were transported in broth media, and subcultured on Bismuth Sulfite medium. Biochemical and serological assays were performed. *fliC-d* specific gene of *S.* Typhi was amplified as described [9].

### 2.3. Antibiogram Assay

The antibiotic susceptibility of isolates was performed as described earlier [9]. Briefly, ampicillin (AMP), chloramphenicol (C), co-trimoxazole (SXT), ciprofloxacin (CIP), levofloxacin (LEV), azithromycin (AZM), imipenem (IMP), ceftriaxone (CRO), aztreonam (ATM), cefepime (FEP), cefixime (CFM), cefoperazone (CFP), cefoxitin (FOX), and nalidixic acid (NA) antibiotic discs (Oxoid, UK) were used. Isolates were lawned equivalent to 0.5 McFarland turbidity standard on Mueller–Hinten agar and incubated overnight at 37 °C. Zone of inhibition was measured and interpreted for each antibiotic [10].

### 2.4. Molecular Detection of Drug Resistance Genes

After screening of the phenotypic resistance profile, DNA was extracted from selected isolates and processed for *parA*, *parC*, *parE*, *gyrA*, *gyrB* (fluoroquinolone resistance genes), *qnrA*, *qnrB*, *qnrC*, *qnrS*, aac(6’)-ib-cr) (plasmid genes), *dfrA7*, *dfrA14*, *cat* (cotrimoxazole and chloramphenicol resistance genes), *bla*OXA, *blaSHV1*, *blaCTX15,* and *blaTEM* (ESBL genes) were screened by using PCR assay as reported [9]. After phenotypic and molecular antibiotic susceptibility, isolates were considered multidrug resistant (MDR), which exhibited resistance to ampicillin, trimethoprim-sulfamethoxazole, and chloramphenicol, while XDR were those isolates that were resistant to ampicillin, trimethoprim-sulfamethoxazole, third-generation cephalosporins, and fluoroquinolones as per the CDC report [11].

### 2.5. Protein Extraction and Quantification

MDR (*n* = 3) and XDR (*n* = 3) *S*. Typhi isolates were selected for protein extraction and quantification [12]. The fresh BHI broth culture was incubated at 37 °C till attainment of optical density (OD) up to 0.9 at 600 nm. One ml bacterial culture with an OD of 0.9 was centrifuged, and the supernatant was discarded. Urea thiourea was added to the pellet and mixed gently. The cell lysate was centrifuged at 12,000 rpm. The supernatant was processed for protein quantification as described by Bradford [13].

### 2.6. SDS PAGE, and Trypsin In-Gel Digestion 

A 10% resolving gel and 4% stacking SDS-PAGE gel were prepared. Equal proteins from each sample were loaded on the gel. Samples were resolved, and the gel was stained for 1 h in Coomassie Blue. The gel was then de-stained in 10% acetic acid for an overnight shaking incubation, and sliced into small bands. The sliced gel bands were processed for trypsin in-gel digestion as described earlier [14]. 

### 2.7. Peptide Sequencing Using Nanoflow LC-LTQ-Orbitrap *MS* Analysis and Mascot Search

A hybrid ion trap mass spectrometer (LC-LTQ-Orbitrap Velos, Thermo Scientific, Carlsbad, CA, USA) was used for the protein’s analysis. The adsorbed peptides were electrically blasted into the mass spectrometer using a laser-pulled tip of a capillary column with silica-based particles (Model P-2000, Sutter Instruments, Novato, CA, USA). The solvent B amount was added. The ten most significant ions from the full MS scan (*m*/*z* 350–1500) were chosen for the MS/MS analysis. The repetition duration for dynamic exclusion was 24 s, while the exclusion duration was 12 s. MDR and XDR biological replicates were processed by LC-MS/MS.

Mascot v 2.3.02 and MaxQuant v1.2 [15] were used to examine the raw M.S. files from the Orbitrap Velos (Computational Systems Biochemistry, Martinsried, Germany). The protein sequence database for *S.* Typhi (CT18) was retrieved from the NCBI database and archived before being used in the current study. The peptide tolerance was set to 4.5 ppm, while the MS/MS fragment tolerance was set to 0.8Da in MaxQuant. The incidence of false discovery might be as high as 1%. The differentially regulated protein’s ratio was derived by dividing the XDR protein by the MDR *S.* Typhi protein; if the ratio of XDR to MDR was greater than two, the protein was considered up-regulated; if it was less than 0.5, the protein was considered down-regulated. Three biological replicate experiments were incorporated.

### 2.8. Data Analysis

Proteins with an average fold change ≥2 and ≤0.5 and a *p*-value of ≤0.05 were statistically considered significant differentially regulated proteins using the student’s *t*-test (Microsoft, Redmond, DC, USA).

### 2.9. Bioinformatics Analysis

In the bioinformatics study, the drug resistance gene sequences were first BLAST and tabulated. The FASTA sequences of significant up-regulated and down-regulated proteins were obtained from Uniport (https://www.uniprot.org/, accessed on 28 June 2023), and archived for further use. For the protein-protein interactions, the STRING v11.5 database was used to upload the up- and down-regulated proteins list, and their interactions were documented [16]. A web browser identified the indicated regulated proteins’ subcellular localization as “Cellugo prediction” (http://cello.life.nctu.edu.tw/cello2go/, accessed on 28 June 2023). For antigenicity predictions, the VaxiJen v2.0 web server (https://www.ddg-pharmfac.net/vaxijen/VaxiJen/VaxiJen.html, accessed on 28 June 2023) has parameters: a 0.4 threshold value, bacteria as an organism, and sequence output [17]. Then, to CARD (https://card.mcmaster.ca, accessed on 28 June 2023), the FASTA sequences of the up- and down-regulated proteins were uploaded to search for the resistome, and their relevance to resistance was already recognized [18]. 

## 3. Results and Discussion

### 3.1. Phenotypic and Genotypic Antibiotic Susceptibility Assay for S. Typhi 

*S.* Typhi was identified using biochemical and PCR-based assays. MDR and XDR *S.* Typhi isolates were confirmed through phenotypic and genotypic antibiotic susceptibility methods (Table 1). 

Previously XDR *S.* Typhi were reported from Pakistan, which were resistant to most of the available antibiotics, thus limiting choice of therapeutic antibiotics [1,3,9]. In the current study, XDR and MDR isolates were selected to process for differential proteomics.

### 3.2. Differential Proteomics of XDR vs. MDR S. Typhi 

Proteomics tools are commonly employed for the detection of biomarkers, the search for vaccine candidates, and for therapeutic drug targets. Several studies have reported the importance of proteome analysis in the molecular pathogenesis of *S.* Typhi [5,6,9].

**Figure 1:**
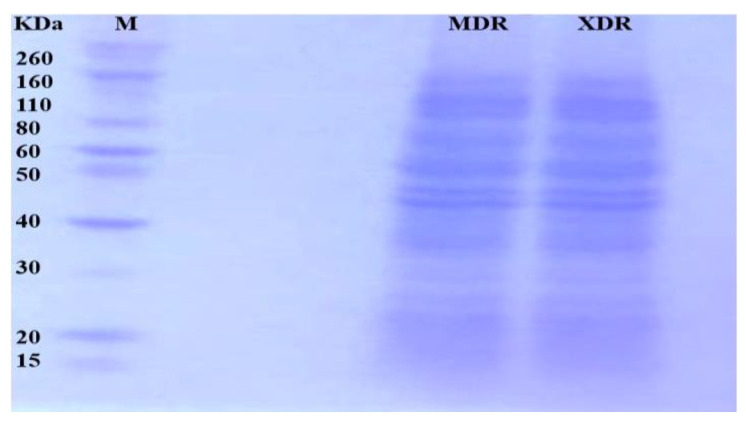
Proteins from MDR and XDR *S.* Typhi isolates: Proteins were extracted, quantified, and loaded on 10% SDS-PAGE. The gel bands were excised for LC MS/MS analysis.

**Figure 2:**
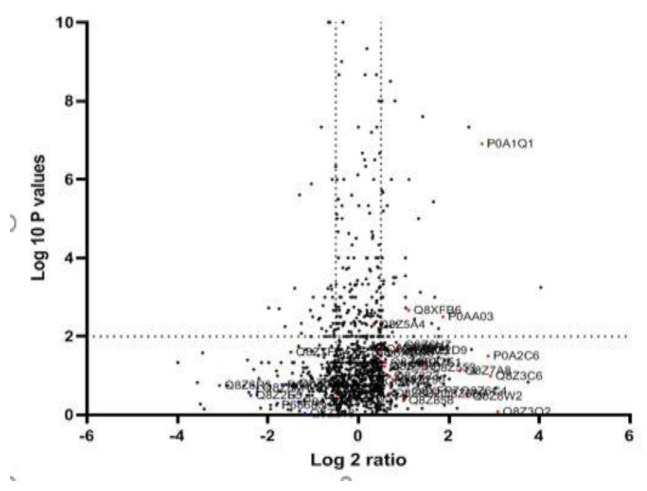
Quantitative proteomic data of XDR and MDR *S.* Typhi: The logarithmic values of the abundance ratios are reported on the x-axis of a protein volcano plot. The negative logarithmic *p*-values calculated from the *t*-test on data from three biological replicates are plotted on the y-axis. Two-fold (horizontal) and *p* < 0.05 cut-offs are indicated by dotted lines (vertical). LC-MS/MS investigations may not be able to differentiate proteins with homologous sequences unless distinct peptides are found.

After MDR and XDR *S.* Typhi confirmation, the protein lysate was resolved on SDS PAGE and stained. Each gel band was sliced and processed for LC-LTQ-Orbitrap *MS* analysis (Figure 1). A total of 1267 proteins were identified. The differential proteome data are plotted on a volcano plot. Two-fold (horizontal) and *p* < 0.05 cut-offs are indicated by dotted vertical lines (Figure 2). 

**Figure 3:**
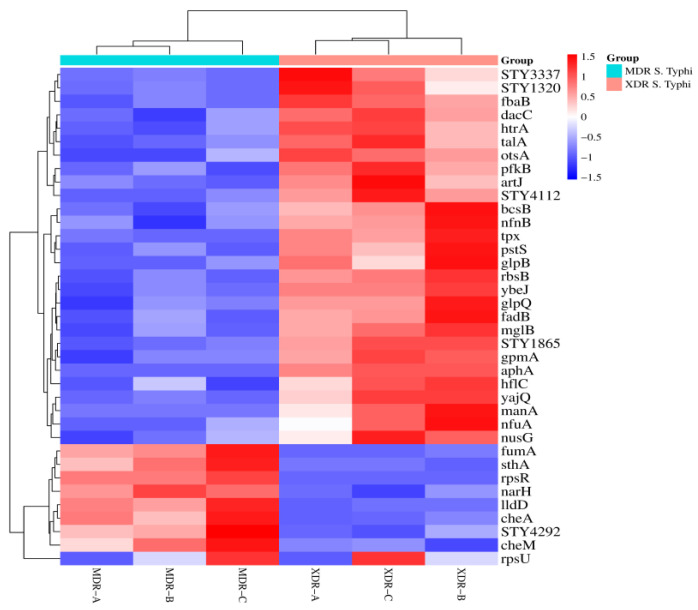
Heat map of XDR and MDR *S.* Typhi: A matrix is created to present the protein expression changes (fold change) in MDR (n = 3) vs. XDR (n = 3) *S.* Typhi isolates. The Euclidean distance method was used. Branching nodes of the heat map represent the inter-cluster dissimilarity. An online resource (https://www.bioinformatics.com.cn/plot_basic_cluster_heatmap_plot_024_en, accessed on 5 January 2024) was used to build a cluster heat map.

The heat map cluster matrix between XDR and MDR *S.* Typhi was created to present the protein expression changes (fold change). The column represents the sample type, while the rows represent proteins (Figure 3).

After data analysis, 28 proteins were significantly up-regulated, while 9 were down-regulated in XDR *S.* Typhi. 28 proteins, including RbsB, STY3337, Tpx, STY1865, PfkB, AphA, ArtJ, YbeJ, MglB, GpmA, YajQ, STY4112, FadB, GlpB, HflC, NfnB, OtsA, GlpQ, NusG, TalA, STY1320, BcsB, NfuA, PstS, FbaB, HtrA, DacC, and ManA, were up-regulated in XDR *S.* Typhi. There were 9 proteins (CheM, SthA, LldD, CheA, NarH, RpsR, FumA, STY4292, and RpsU) significantly down-regulated in XDR *S.* Typhi (Table 2).

In the up-regulated proteins, RbsB is a component of the ABC transporter complex (RbsABC) that deals with ribose import, and the PstS protein deals with phosphate import. These periplasmic proteins were up-regulated at high fructose concentrations in Streptomyces lividans [19]. The up-regulation of RbsB and PstS might have a role in the emergence of the XDR *S.* Typhi phenotype.

Tpx, HtrA, and ArtJ were stress-response proteins that were up-regulated. Tpx is a thiol peroxidase and plays a role in stress response and metabolism [20]. HtrA is a heat shock protein; the deletion of hfq resulted in increased HtrA protein levels [19]. MglB is a chemotaxis-related up-regulated protein. In *S.* Typhimurium, the protein MglB is also overexpressed and engaged in chemotaxis [20]. The three proteins that have been up-regulated serve a variety of purposes, including those of coenzymes (AphA and DacC) and virulence proteins (VirP) (HlfC). AphA is an acid phosphatase that causes the dephosphorylation of several metabolic molecules. The protein DacC facilitates the production of peptidoglycans. In *S.* Typhimurium, the protein HlfC is part of a hfq operon and is essential for virulence [21].

Two proteins (CheA and CheM) involved in chemotaxis (bacterial movement) were down-regulated. In a previous study, these proteins were variably expressed. In the down-regulated protein, RpsU is a growth protein, and, when the growth rate in bacteria decreased, its gene was reported as a constituently expressed [20].

Among the other down-regulated proteins, LldD, RpsR, and FumA, among others, were reported to be involved in metabolic pathways. L-lactate is converted to pyruvate by the L-lactate dehydrogenase protein (LldD) [22]. The RpsR protein stabilizes the 30S subunit by attaching to protein S6. In aerobic circumstances in *E. coli*, fumarate dehydrogenase protein (FumA) is down-regulated, whereas its gene, FumA, is reported to be up-regulated [23]. 

### 3.3. Prediction of Cellular Localization, Antigenicity, and Resistome Map of Differentially Regulated Proteins

Among the 28 up-regulated proteins of XDR *S.* Typhi, the localization prediction tool showed proteins of the cytoplasmic (*n* = 17), periplasmic (*n* = 14), and inner membrane (*n* = 9). We found that 23 proteins were predicted to be antigenic, and 5 were regarded as non-antigenic. Most of the proteins (*n* = 24) were matched with the resistome map. Among the down-regulated proteins, 7 were cytoplasmic and 4 were inner membrane. Similarly, 8 proteins were antigenic, and all the proteins matched the resistome map (Table 2).

The resistome map of the regulated proteins showed a relationship with predicted antibiotic resistance mechanisms. It is evident that AMR comes with a survival strategy that may alter bacterial growth. To compensate for the survival cost or to handle the stress posed by antibiotics, the bacteria tend to fine-tune nutrient demands [24]. The possible explanation for the altered XDR associated proteins might be due to selective antibiotic pressure to manage the toxic effects of antimicrobial drugs.

### 3.4. Annotation of the Biological Function of Identified Proteins

The up-regulated proteins were mostly involved in the metabolic pathways (*n* = 10), biosynthesis of secondary metabolites (*n* = 8), and ABC transporters (*n* = 5). In the down-regulated proteins, most were involved in the metabolic pathways (*n* = 4) (Table 3). 

The proteome profile of XDR *S.* Typhi reflects that the up-regulated and down-regulated proteins are involved in diverse functions, not limited to ABC transport, metabolism, respiration, and signal transduction.

### 3.5. Protein–Protein Interaction and Annotation by STRING

**Figure 4:**
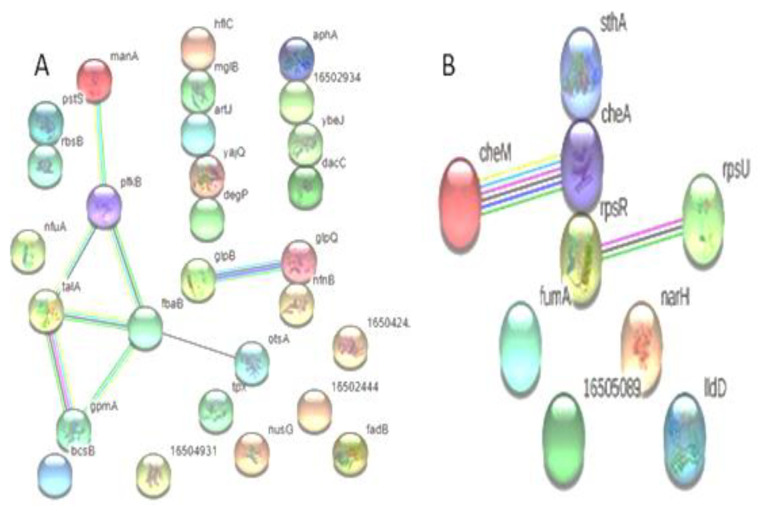
Predicted interaction of up-regulated proteins (**A**) (*n* = 28) and down-regulated proteins (**B**) (*n* = 9) of *S.* Typhi: (**A**) Showing direct interaction of hflC, mglB, artJ, yajQ, degP, gloQ, nfnB, pstS, rbsB, aphA, 16502934, ybeJ, and dacC and indirect interaction of manA, pfkB, talA, fbaB, gpmA, otsA, glpB, and gloQ. (**B**) Showed the non-interaction of IIdD, narH, 16505089, fumA, and cheA and the direct interaction of rpsR, cheA, and sthA, and the indirect interaction of cheM with cheA; rpsR interacts with rpsU.

A total of 28 proteins that are up-regulated in XDR are included in the protein–protein interaction map. *S.* Typhi demonstrated that most proteins interacted with other proteins both directly (*n* = 13) and indirectly (*n* = 8). Among the down-regulated proteins, three showed direct interaction, while four exhibited indirect interaction with other proteins (Figure 4).

There is a growing body of literature on the role of protein–protein interactions in biological functions. Protein interactions are not limited to enzyme functions, metabolic reactions, cascade pathways, and negative and positive regulation [25]. In the present study, protein interaction prediction analysis showed direct interaction. These proteins are involved in several biological processes and might have a role in the resistance mechanisms of MDR and XDR *S.* Typhi. 

Overall, the expression of these proteins might trigger the AMR mechanism that leads to increased resistance in *S.* Typhi. Further characterization of regulated proteins would be helpful to understand the XDR *S.* Typhi response and adaptation to available antibiotics, and will explore new avenues in the fight against global antimicrobial resistance.

## 4. Conclusions

The proteome profile of XDR *S.* Typhi identified 28 up-regulated and 9 down-regulated proteins. Functional annotation, interaction, localization, antigenicity, and resistome prediction further highlighted their possible role in the AMR mechanisms among XDR *S.* Typhi. 

## Figures and Tables

**Table 1:** Antibiotic susceptibility profile of MDR and XDR *S.* Typhi isolates processed for proteome analysis.

Phenotypic Resistance Profile															Genotypic Resistance Profile
S. No.	AMP	C	SXT	CIP	LEV	AZM	IMP	CRO	ATM	FEP	CFM	CFP	FOX	NA	Genes Detected
MDR isolates															
1.	R	R	R	I	I	S	S	S	S	S	S	S	S	R	*aac(6’)-lb-cr*, *gyr A*, *gyrB*, *parC*, *parE*, *qnrS*, *qnrA*, *qnrC*, *qnrB*, *cat*
2.	R	R	R	S	S	S	S	S	S	S	S	S	S	R	*aac(6’)-lb-cr*, *gyr A*, *gyrB*, *parC*, *parE*, *qnrS*, *qnrA*, *qnrC*, *qnrB*, *cat*
3.	R	R	R	I	I	S	S	S	S	S	S	S	S	R	*aac(6’)-lb-cr*, *gyr A*, *gyrB*, *parC*, *parE*, *qnrS*, *qnrA*, *qnrC*, *qnrB*, *cat*
XDR isolates															
1.	R	R	R	R	R	S	S	R	R	R	R	R	R	R	*aac(6’)-lb-cr*, *gyr A*, *gyrB*, *parC*, *parE*, *qnrS*, *qnrA*, *qnrC*, *qnrB*, *cat blaTEM*, *blaCTX15*, *blaSHV1*, *blaOXA*
2.	R	R	R	R	R	S	S	R	R	R	R	R	R	R	*aac(6’)-lb-cr*, *gyr A*, *gyrB*, *parC*, *parE*, *qnrS*, *qnrA*, *qnrC*, *qnrB*, *cat blaTEM*, *blaCTX15*, *blaSHV1*, *blaOXA*
3.	R	R	R	R	R	S	S	R	R	R	R	R	R	R	*aac(6’)-lb-cr*, *gyr A*, *gyrB*, *parC*, *parE*, *qnrS*, *qnrA*, *qnrC*, *qnrB*, *cat blaTEM*, *blaCTX15*, *blaSHV1*, *blaOXA*

AMP: ampicillin, C: chloramphenicol, SXT: co-trimoxazole, CIP: ciprofloxacin, LEV: levofloxacin, AZM: azithromycin, IMP: imipenem, CRO: ceftriaxone, ATM: aztreonam, FEP: cefepime, CFM: cefixime, CFP: cefoperazone, FOX: cefoxitin, NA: nalidixic acid. Fluoroquinolone resistance genes: *parA*, *parC*, *pare*, *gyr A*, and *gyrB*. Plasmid genes: *qnrA*, *qnrB*, *qnrC*, *qnrS*, and aac(6’)-ib-cr). Cotrimoxazole and chloramphenicol resistance genes*: dfrA7*, *dfrA14*, *cat*. ESBLgenes: * bla*OXA, *blaSHV1*, *blaCTX15*, and *blaTEM*.

**Table 2:** Differentially regulated proteins of *XDR* *S.* Typhi with gene names, functions, subcellular location (threshold 0.001), antigenicity (VaxiJen, threshold 0.4), and resistome match.

S. No.	Accession	Gene	Function	Location	Antigenicity	Resistome Match
Up-regulated proteins						
1	Q8Z8W2	*yajQ*	Not known	Cytoplasm	Antigen	Mutant Elongation Factor Tu of *E. coli* conferring resistance of kirromycin.
2	Q8Z3Q2	*STY3337*	Not known	Cytoplasm	Antigen	LpsB (lipopolysacahiride synthesis) of *A*. *baumannii*, and macrolide2’-phosphotransferase of *Brachybacterium faecium.* TEM-22, TEM-125, TEM-78, TEM-185, and TEM-79 of *E. coli*.
3	Q8Z2D9	*STY4112*	Glutathione metabolism	Cytoplasm and inner-membrane	Non-Antigen	oqxB of *Escherichia coli* belongs to resistance-nodulation-division (RND) efflux pump conferring resistance to fluoroquinolone.
4	Q8Z6C4	*STY1865*	Trans-membrane protein	Periplasm, inner-membrane and cytoplasm	Antigen	ERP-1 of *Erwinia persicina* is a class A beta-lactamase.
5	Q8Z6H7	*pfkB*	Fructose metabolism	Inner-membrane and cytoplasm	Antigen	The gyrA of *Haemophilus parainfluenzae T3T1* confer resistance to fluoroquinolones.
6	Q8Z3C6	*fadB*	Fatty acid metabolism.	Cytoplasm and inner membrane	Non-antigen	mtrE of *Neisseria gonorrhoeae* coding an outer membrane exporter protein belongs to RND. The plasmids and integrons coded AAC (6’)-Iq (aminoglycoside acetyltransferase) of *Klebsiella pneumoniae*.
7	Q8Z553	*glpB*	Carbohydrate metabolism	Inner-membrane	Antigen	All tetracyclines including tigecycline, eravacycline, and omadacycline are inactivated by tetX, Tet(X4), and Tet(X3) genes of *Bacteroides fragilis*, *E. coli*, *A. baumannii*, and *A. pittii,* respectively.
8.	P0A1Q1	*otsA*	In osmoprotection	Cytoplasm	Antigen	In the BaeSR regulatory system, BaeS is a sensor kinase and BaeS phosphorylates BaeR to boost its activity, although overexpressed BaeR does not require it to impart resistance. CTX-M beta-lactamase confer resistance to cephalosporins
9	Q8Z7E1	*STY1320*	Not known	periplasm and cytoplasm	Antigen	BES-1, R39, DES-1, SHV-41, GES-8, and GES-15 are beta lactamases of *Serratia marcescens*, *Actinomadura* spp., *Desulfovibrio desulfuricans K. pneumoniae*, and *P. aeruginosa*.
10	Q8Z290	*bcsB*	Binds bis-(3’-5’) cyclic diguanylic acid	Inner-membrane	Antigen	The protein Q8Z290 showed resemblance to extended extended-spectrum beta lactamase TEM-138, TEM-188 and TEM-1 found in *Salmonella enterica*.
11	Q8Z858	*dacC*	Cell wall synthesis.	Periplasm, extra-cellular and periplasm	Antigen	CMY-145, OXA-198 and SHV-156, beta-lactamase, of Escherichia coli and P. aeruginosa, respectively, conferring resistance to cephamycin. APH (3’)-IIb, chromosomal-encoded aminoglycoside phosphotransferase of *P. aeruginosa.*
12	Q8Z556	*glpQ*	Lipid metabolism	Periplasm and inner-membrane	Antigen	No resistome matched.
13	Q8Z6R4	*manA*	Not known	Periplasm, inner-membrane and cytoplasm	Antigen	A multidrug efflux complex, AdeABC, an outer membrane factor, AdeC.
14	Q8Z223	*nfuA*	Iron-sulfur biogenesis	Cytoplasm	Antigen	vanF of *Paenibacillus popilliae* ATCC 14706, vanI of *Desulfitobacterium hafniense*, vanB of *Enterococcus faecium* and confer resistance to vancomycin.
15	Q8Z7A8	*Tpx*	Antioxidative stress	Cytoplasm and periplasm	Non-Antigen	gyrB of *Mycobacterium leprae* by point mutation results in fluoroquinolone resistance.
16	P0A2C6	*rbsB*	ABC transporter	Periplasm	Antigen	vanXD of *Enterococcus faecium*. OXA-299 of *Acinetobacter* spp. MexQ of *Pseudomonas aeruginosa*. BRO-2, beta-lactamase, of *Moraxella catarrhalis*.
17	Q8XGB6	*hflC*	Protease regulator	Cytoplasm and inner membrane	Antigen	*gyrA* of *Clostridioides difficile*, tetB (60) is a subunit of tetAB (60) imparting resistance to tetracycline and tigercycline, In *Stenotrophomonas maltophilia*, smeB is the inner membrane multidrug exporter of the efflux complex smeABC.
18	Q8Z8M5	*nfnB*	Not known	Periplasm and cytoplasm	Antigen	*Bla1* of *Bacillus anthracis* is beta lactamase. *rpoC* of *Clostridioides difficile* 630 *patA* of *Streptococcus pneumoniae* is an ABC transporter’s part and along with *PatB* confer fluoroquinolone resistance.
19	O08430	*aphA*	Dephosphorylase	Periplasm	Antigen	Tet (55) conferring resistance to tetracycline by inactivating flavoenzyme. emrK of *Escherichia coli* belongs to MFS efflux pump mediates multidrug efflux.
20	Q8Z2P5	*pstS*	Phosphate import	Periplasm	Antigen	No resistome matched.
21	Q8XFP7	*fbaB*	No known function	Cytoplasm, outer-membrane, periplasm	Non-antigen	No resistome matched.
22	P0AA03	*nusG*	Transcription	Cytoplasm	Antigen	No resistome matched.
23	Q8Z5A4	*mglB*	Transports carbohydrate	Periplasm	Antigen	*mecA* of *Staphylococcus aureus* confer resistance against drug class penum.
24	Q8Z8B2	*gpmA*	phosphoglycerate synthesis	Cytoplasm	Non antigen	vanN of *Enterococcus faecium*; JOHN-1 of *Flavobacterium johnsoniae*.
25	Q8XFB6	*artJ*	Amino acid transport	Periplasm	Antigen	AAC (3)-IId, and AAC (3)-IIe (aminoglycoside acetyltransferase) of *E. coli.*
26	Q8Z8G8	*ybeJ*	Not yet known	Periplasmic	Antigen	AER-1 of *Aeromonas hydrophila* and conferring resistance to cephalosporins.
27	Q8Z4T0	*talA*	Pentose-phosphate pathway	Cytoplasm	Antigen	PmrF of *Pseudomonas aeruginosa* is necessary for the production and transferring of 4-amino-4-deoxy-L-arabinose (Ara4N) to Lipid A.
28	Q8Z9B0	*htrA*	Protein metabolism	Periplasm	Antigen	AAC (3)-VIIa of *Streptomyces rimosus* is a chromosomal-encoded aminoglycoside acetyltransferase conferring resistance against aminoglycosidic antibiotics.
Down-regulated proteins						
1	P68683	*rpsU*	30S ribosomal subunit	Cytoplasm	Antigen	Haemophilus parainfluenzae parC, and parE of *Salmonella serovars*.
2	Q8Z2E5	*lldD*	Carbohydrate metabolism	Inner-membrane and cytoplasm	Antigen	*Mycobacterium tuberculosis* tlyA. Tet(T) of *Streptococcus pyogenes.*
3	Q8Z5U8	*cheA*	Signal transduction	Cytoplasm	Antigen	EvgS of Escherichia coli phosphorylates; EvgA, and ArlS phosphorylates; LiaS of *Enterococcus faecalis*, and CpxA of *Escherichia coli*.
4	P0A7U1	*rpsR*	Protein turnover	Inner-membrane	Non-antigen	NmcR of *Enterobacter cloacaeis* a homolog of the LysR regulator contributing the regulation of NmcA beta-lactamase.
5	Q8Z218	*STY4292*	Not known	Cytoplasm	Antigen	AAC(3)-IIa (aminoglycoside acetyltransferase) of *K. pneumoniae*, *E. cloacae*, *Actinobacillus pleuropneumoniae*, *S. typhimurium*, *Citrobacter freundii*, and *P. aeruginosa.*
6	Q8Z6R5	*fumA*	Carbohydrate metabolism	Cytoplasm	Antigen	PatB of *Streptococcus pneumoniae*.
7	Q8Z7F8	*narH*	Nitrate reductase activity	Cytoplasm	Antigen	rpld of *Neisseria gonorrhoeae.*
8	Q8Z3M1	*cheM*	Not known	Inner-membrane	Antigen	AdeJ is an RND efflux protein that functions as the AdeIJK efflux complex’s inner membrane transporter.
9	P66009	*sthA*	Energy synthesis.	Cytoplasm	Antigen	EreB of *Escherichia coli*, gyrA of *Clostridioides difficile*.

**Table 3:** Functional annotation for the differentially regulated proteins of XDR *S.* Typhi.

ANNOTATED PATHWAY (KEGG) NAME	Proteins Involved
Up-regulated proteins	
Metabolic pathways	ManA, FadB, DacC, GpmA, AphA, PfkB, TalA, FbaB, OtsA, 16504242
Pentose phosphate pathway	PfkB, TalA, FbaB
Methane metabolism	GpmA, PfkB, FbaB
Biosynthesis of amino acids	GpmA, PfkB, TalA, FbaB
ABC transporters	PstS, YbeJ, MglB, RbsB, ArtJ
Carbon metabolism	FadB, GpmA, PfkB, TalA, FbaB
Microbial metabolism in diverse environments	FadB, GpmA, PfkB, NfnB, TalA, FbaB
Biosynthesis of secondary metabolites	ManA, FadB, GlpB, GpmA, PfkB, TalA, FbaB, OtsA
Glycolysis/Gluconeogenesis	GpmA, PfkB, FbaB
Fructose and mannose metabolism	ManA, PfkB, FbaB
Down-regulated proteins	
Microbial metabolism in diverse environments	NarH FumA
Ribosome	RpsR RpsU
Pyruvate metabolism	FumA LldD
Bacterial chemotaxis	CheM CheA
Two-component system	CheM NarH CheA
Metabolic pathways	NarH FumA LldD SthA

## Data Availability

Data will be made available upon request.
